# Roles of RNA Sensors in Host Innate Response to Influenza Virus and Coronavirus Infections

**DOI:** 10.3390/ijms23158285

**Published:** 2022-07-27

**Authors:** Wei Li, Hongnuan Wang, Shijun J. Zheng

**Affiliations:** 1 Key Laboratory of Animal Epidemiology of the Ministry of Agriculture, College of Veterinary Medicine, China Agricultural University, Beijing 100193, China; liwei19940506@163.com (W.L.); whongnuan@163.com (H.W.); 2 Department of Preventive Veterinary Medicine, College of Veterinary Medicine, China Agricultural University, Beijing 100193, China

**Keywords:** influenza virus, coronavirus, RNA sensors, innate immune response, viral RNA

## Abstract

Influenza virus and coronavirus are two important respiratory viruses, which often cause serious respiratory diseases in humans and animals after infection. In recent years, highly pathogenic avian influenza virus (HPAIV) and SARS-CoV-2 have become major pathogens causing respiratory diseases in humans. Thus, an in-depth understanding of the relationship between viral infection and host innate immunity is particularly important to the stipulation of effective control strategies. As the first line of defense against pathogens infection, innate immunity not only acts as a natural physiological barrier, but also eliminates pathogens through the production of interferon (IFN), the formation of inflammasomes, and the production of pro-inflammatory cytokines. In this process, the recognition of viral pathogen-associated molecular patterns (PAMPs) by host pattern recognition receptors (PRRs) is the initiation and the most important part of the innate immune response. In this review, we summarize the roles of RNA sensors in the host innate immune response to influenza virus and coronavirus infections in different species, with a particular focus on innate immune recognition of viral nucleic acids in host cells, which will help to develop an effective strategy for the control of respiratory infectious diseases.

## 1. Introduction

Innate immunity is the natural resistance of the host against pathogens, including viruses, bacteria, fungi, and others. The specific components of pathogens including nucleic acids, proteins, peptidoglycan, and so on, which are called pathogen-associated molecular patterns (PAMPs), can be recognized by the sensors of host cells that are called pattern recognition receptors (PRRs) to initiate the innate immune response. Up to now, at least five groups of PRRs have been identified, including toll-like receptors (TLRs), nucleotide-binding oligomerization domain-like receptors (NLRs), retinoic acid-inducible gene I-like helicases receptors (RLRs), c-type lectin receptors (CLRs), and DNA sensors including AIM2-like receptors (ALRs) and Cyclic-GMP-AMP Synthase (cGAS). These PRRs have different structures and are located differently in host cells. Upon engagement of PAMPs by host PRRs, the signal-transducing pathway is initiated with the recruitment of adaptors to the activating domains of PRRs, ultimately eliciting the innate immune response.

As the transmembrane proteins, TLRs recognize the nucleic acids, lipoproteins, and lipopolysaccharides (LPS) of pathogens [1,2,3,4]. The engagement of TLRs with PAMPs leads to the recruitment and activation of downstream adapters, such as myeloid differentiation factor 88 (MyD88), TIR domain-containing adapter-inducing interferon-β (TRIF), and TIR domain-containing adapter protein (TIRAP). These adapters are involved in NF-κB, IRF, or AP-1 signaling pathways to trigger innate immune responses [5,6,7]. RLRs, mainly existing in the cytoplasm, consisting of several members, such as retinoic acid-inducible gene I (RIG-I), melanoma differentiation-associated gene 5 (MDA5), and laboratory of genetics and physiology 2 (LGP2), sensing the pathogenic double-stranded RNA (dsRNA)to activate the mitochondrial antiviral signaling protein (MVAS) through the unique CARD domain, and initiating NF-κB and IRFs signaling pathways [8,9,10,11,12,13]. NLRs, a member of PRRs in the cytoplasm, primarily sense certain components of bacteria. Among them, the most important representatives are NOD1 and NOD2. Once the bacterial peptidoglycan (PGN) is recognized by NOD1/NOD2, the NF-κB and IRFs signaling pathways are subsequently activated by recruiting and activating the receptor interacting protein 2 (RIP2), triggering an innate immune response [14,15,16]. The engagement of NLRs by PAMPs of bacteria or damage-associated molecular patterns (DAMPs) also induces the formation of inflammasomes, which activate pro-caspase-1 to cleave the gasdermin family proteins, resulting in pyroptosis and inflammatory response [17,18,19]. CLRs are cell membrane receptors that recognize fungi [20]. Once sensing appropriate ligands, CLRs will act on MALT1/Bcl-10 by activating spleen tyrosine kinase (Syk) through its ITAM domain, and finally activate NF-κB and AP-1 signaling pathways [21]. In addition, the activated Syk can slao induce the innate immune response by activating the NF-AT signaling pathway [22]. Some cytoplasmic or nuclear DNA receptors (cGAS, IFI16, AIM2, DAI, etc.) discovered in recent years are capable of specifically recognizing exogenous DNA. On the one hand, these DNA receptors induce the oligomerization of STING to induce the innate immune response through a series of signal transduction pathways [23,24,25]. On the other hand, similar to NLRs, they also induce the formation of inflammasomes and cause a strong inflammatory response [26,27]. Furthermore, it was reported that type III interferon (IFN-III) can also be produced in mucosal tissue cells through a similar pathway to IFN-I and demonstrate an intense antiviral activity [28,29,30,31]. A cytoplasmic DNA sensor ku70, which specifically induces IFN-III response, has been discovered recently [32]. After recognizing exogenous DNA, Ku70 translocates from the nucleus into the cytoplasm and induces the production of IFN-III through the STING-IRF1/7 signaling pathway, indicating a novel mechanism to initiate innate immune response [33,34,35].

Viral respiratory diseases have always been serious threats to human health. After infection, the patients usually display fever, sore throat, cough, and wheezing. In severe cases, such as HPAIV, SARS-CoV, and SARS-CoV-2 infections, patients usually display more severe systemic inflammatory responses or even succumb to death. With the outbreak of SARS-CoV-2 in late 2019, a growing number of studies have focused on viral respiratory diseases. This review is mainly focused on the influenza virus and coronavirus, two commonly observed respiratory viruses in terms of their intracellular RNA sensor-mediated innate immune responses following infection.

## 2. Influenza Virus

Influenza virus is a segmented single-stranded negative RNA virus of *Orthomyxoviridae* with high variability and transmissibility [36]. Segments of the viral genome encode 10 major proteins, among which two viral surface glycoproteins, hemagglutinin (HA) and neuraminidase (NA), are mainly used as the basis for viral typing [37,38,39,40]. Influenza virus is one of the most causative pathogens of human respiratory diseases, causing seasonal influenza and occasional influenza pandemics every year. Influenza viruses can be divided into four types: influenza A virus (IAV), influenza B virus (IBV), influenza C virus (ICV), and influenza D virus (IDV). Among them, IAV infects humans and a variety of animals, such as chickens, pigs, horses, marine mammals, etc, causing zoonotic diseases. H5N1, H7N9, and H1N1, the members of IAV that mainly infect poultry or mammals, pose a serious threat to public health. Considering that IAV is a segmented RNA virus, antigenic drift and antigenic shift may occur all the time during its replication due to the poor fidelity of RNA polymerase so that new subtypes of IAV could randomly and unexpectedly emerge. The new subtypes of IAV that better escape the host response would become dominant, and even break the interspecific barrier, resulting in cross-species transmission in humans [41]. Specifically, on the one hand, the IAV protein with accumulated amino acid mutations, especially NS1 [42] and PA-X [43], plays a major role in contributing to the evolution of IAV by inhibiting the host’s innate immune response through a variety of mechanisms: (i) Viral proteins directly interact with PRRs or innate immune molecules to inhibit the activation of the downstream signaling pathway (shown in Table 1) [44,45]; (ii) viral proteins bind to the host proteins [46,47] or dsDNA [48] to suppress the expression of antiviral genes; (iii) viral proteins bind to viral dsRNA to evade the innate immune recognition by RNA sensors [49]. On the other hand, the random reassortment of genome segments between different strains directly contributes to viral constant evolution and the generation of new subtypes [50,51]. It was reported that PB2 and PA gene segments containing mammalian adaptive mutations of H9N2 could be reassorted into the cocirculating H7N9 virus, resulting in the emergence of a new H7N9 virus genotype [52]. Thus, an in-depth understanding of the innate immune response caused by IAV RNA in different species would help to provide theoretical guidance for the control of IAV.

### 2.1. The Innate Immune Response to IAV Infection in Humans

In humans, viral RNA is primarily sensed by three types of PRRs, namely TLRs, RLRs, and NLRs [53,54,55]. Among them, TLRs are mainly responsible for the recognition of viral RNA in the endosome. Specifically, TLR3 senses dsRNA, while TLR7 and TLR8 sense single-stranded RNA (ssRNA) [56,57,58]. TLR9 recognizes CpG DNA or viral nucleic acids [59]. RLRs recognize dsDNA in the cytoplasm [60]. In addition, NLRs may also sense viral RNA, and then lead to the formation of inflammasomes [61,62].

It was reported that IAV replication intermediates could be recognized by TLR3 in human respiratory epithelial cells, which recruits TRIF and PI3K adapters and further activates IRFs and NF-κB signaling pathways [63,64]. In vivo, TLR3^−/−^ mice displayed a higher viral titer and fewer inflammatory mediators upon H3N2 infection than wild-type (WT) mice, indicating the crucial role of TLR3 in resisting IAV infection [53,65]. However, despite the presence of TLR3, WT mice still show higher mortality, which may be attributable to their excessive inflammatory response [53]. Notably, the innate immune response mediated by TLR3 appears to be more inclined to induce the production of inflammatory cytokines rather than IFNs [66]. For IAV ssRNA, it can be sensed by TLR7 to activate NF-κB in a MyD88-dependent signaling pathway in pDCs, which contributes to the production of IFN-I [67,68]. Furthermore, the essential role of TLR7 in inflammatory cytokine activation was determined in TLR7^−/−^ mice [69]. Although a few reports demonstrated that TLR8 was activated upon IAV infection [70,71], the mechanism by which TLR8 drives the innate immune response is slightly distinct from TLR7. TLR7 mainly induces the expression of Th17-polarizing cytokines, while TLR8 is closely related to the expression of Th1-type cytokines and IFN-I in CD14+ monocytes upon IAV infection [70], which suggests that TLR7 and TLR8 activate distinct pathways in monocytes. Moreover, TLR7 seems to play a role in the recognition of ssRNA to induce the IFN-I expression in dendritic cells, while TLR8 prefers to sense IAV ssRNA to induce the IL-12 expression in monocytes [71]. Thus, it is necessary to explore whether TLR7 or TLR8 has a cell-type preference upon IAV infection. It was found that the expression of TLR9 significantly increased upon H1N1 infection, suggesting that TLR9 may also be involved in the innate immune response against H1N1 infection [72].

In addition to TLRs, RLRs, as classical cytoplasmic RNA sensors, also play an important role in sensing viral RNA and combating viral infection [73,74]. It was reported that RIG-I was activated and engaged to MVAS on mitochondria upon IAV infection, initiating the expression of inflammatory cytokines and IFN-α/β in human alveolar epithelial cells [64,75]. It was also found that RIG-I could further enhance the expression of IFN-I through the MAVS-PI3K signaling pathway after recognizing the accumulated vRNA of IAV [76]. Furthermore, a recent study showed that RIG-I was present not only in the cytoplasm, but also in the nucleus [77]. The presence of RIG-I in the nucleus mainly senses vRNP in the process of IAV replication and induces the innate immune response, but the specific mechanism needs to be further investigated. However, it is still controversial whether MDA5, another representative member of RLR, contributes to the engagement of IAV dsRNA. Previous studies indicated that MDA5 was mainly involved in the innate immune response caused by some picornaviruses rather than IAV [78,79]. It was found that RIG-I acted as a primary PRR for IAV dsRNA in primary murine embryonic fibroblasts (MEFs), while MDA5 mainly functions as a transcriptional inducer, which benefits the amplification of ISG production [80]. In brief, MDA5 does not directly contribute to the engagement of IAV RNA, but it may participate in the amplification of host antiviral response by degrading viral nucleic acids through the OAS/RNase L system [80]. In addition, the length and structure of foreign RNA may also contribute to the differences in RNA recognition between RIG-I and MDA5. MDA5 mainly recognizes long dsRNA (>1 kb) and prefers to sense mRNA lacking ribose 2′-O methylation in the 5′cap structure, while RIG-I prefers to recognize short dsRNA sequences (<1 kb) [60,81,82]. Thus, more efforts will be required to investigate whether MDA5 is involved in IAV RNA recognition. Recently, it was found that IFI16, a well-known DNA receptor, also recognizes IAV RNA through the H1Na domain, eliciting an innate immune response by binding to RIG-I through another PYRIN domain to promote the activation of RIG-I [83]. It seems that our current understanding of nucleic acid receptors is quite limited, and there may be more nucleic acid sensors that can recognize DNA as well as RNA. In addition to IFN-I, there is no doubt that IFN-λ, a core molecule of innate mucosal immunity, is also abundantly expressed upon IAV infection [84,85,86,87]. However, the upstream signal transduction mechanisms of IFN-I and IFN-III are slightly different. IRFs play a dominant role in IFN-α/β expression, while NF-κB seems to be a key regulator of IFN-λ expression [88]. Furthermore, the IFN-β expression depends on the coordinated functions of a multifactor enhanceosome and the IFN-α expression on the IRF-binding cis-promoter elements, while the expression of IFN-III relies on the activation of NF-κB or IRFs alone, which suggests that there is a wider range of stimuli in inducing IFN-III expression [87,89,90]. These findings were further confirmed at the level of single-cell [91]. Although the sequence and structure between IFN-I receptors (IFNAR1/IFNAR2) and IFN-III receptors (IFNLR1/IL10R2) may vary, the downstream signal transduction process is highly similar, in which both trigger the expression of interferon stimulated gene (ISG) through the JAK-STAT signaling pathway [92,93].

**Figure 1:**
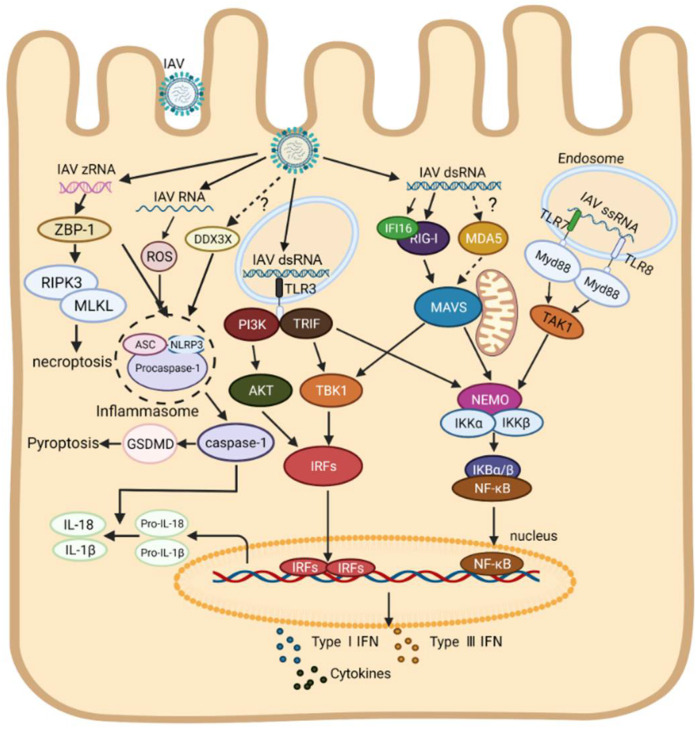
Schematic diagram of innate immune response caused by IAV RNA. Upon IAV infection, viral ssRNA in the endosome is sensed by TLR7/8 and recruits the MyD88 adapter. Activated MyD88 induces the production of inflammatory cytokines through the TAK1-IKK-NF-κB signaling pathway. Viral double-stranded RNA in the endosome is sensed by TLR3, which further recruits TRIF and PI3K adapters, inducing the production of IFN through the TAK1-IRFs signaling pathway. Viral dsRNA in the cytoplasm is sensed by RIG-I and IFI16, leading to activation of NF-κB and phosphorylation of IRFs by binding to MAVS on mitochondria. Furthermore, IAV RNA can be sensed by NLRP3, which promotes the formation of inflammasomes, causing pyroptosis and secreting the IL-1β and IL-18. Of note, there is no direct evidence to demonstrate that IAV RNA can be recognized by DDX3X and MDA5 in mammals, which is indicated by dotted lines. Abbreviation: ROS, reactive oxygen species; MLKL, mixed lineage kinase domain-like pseudokinase. Other abbreviations are shown in the legend to Figure 2.

In recent years, the research on inflammasomes has become a hot area. It is now believed that two pathways concern the formation of inflammasomes, including the classical pathway mediated by caspase-1 and the non-classical pathway by caspase-11 [94,95,96,97]. It was reported that the expressions of NLRP3, caspase-1, pro-IL-1β, and pro-IL-18 were up-regulated via the NF-κB signaling pathway upon IAV infection, inducing the formation of inflammasomes in a caspase-1-dependent pathway [68]. IAV RNA or poly (I:C) can also be directly sensed by NLRP3 in human macrophages, resulting in pyroptosis and secreting a number of mature IL-1β and IL-18 [55,98,99]. Thus, it is very likely that NLRP3 acts as an intracellular RNA sensor for IAV to induce the formation of inflammasomes as well as inflammatory responses. Furthermore, a recent study indicates that DEAD-Box Helicase 3X (DDX3X), a member of the RNA helicase family protein, was also involved in the formation of NLRP3 inflammasome during WT IAV infection, but the specific ligands of DDX3X and related regulatory mechanisms remain unknown [100]. In addition to several known members in the caspase family (caspases-1, 4 and 11), more and more caspases have been shown to contribute to inflammasome formation. For instance, activated caspase-8 cleaves gasdermin D (GSDMD) during Yersinia infection, causing the formation of inflammasome [101,102], and activated caspase-3 can cleave GSDME, inducing pyroptosis and lung necrosis [103], and activated caspase-6 promotes inflammasome formation though activating the ZBP1-RIPK3 complex and induces a strong inflammatory response upon IAV infection [104]. ZBP-1, known as an intracellular dsDNA sensor, was reported to regulate NLRP3 inflammasome activation upon IAV infection [105]. Recently, it was found that ZBP-1 could sense IAV Z-RNA, which promotes the activation of mixed lineage kinase domain-like pseudokinase (MLKL) mediated by RIPK-3, resulting in necroptosis [106]. It seems that ZBP-1 may serve as a new PRR for IAV RNA inhost, which furthered our understanding of the mechanism of cell death and innate immune response caused by IAV RNA. As shown in Figure 1, the RNA sensors-mediated innate immune signal transduction pathways to IAV infection in human cells are summarized (Figure 1).

### 2.2. The Innate Immune Response to IAV Infection in Chickens

Since the avian is one of the main hosts of IAV infection, the IAV from the avian is usually described as the avian influenza virus (avian IAV). Avian IAV is divided into HPAIV and low pathogenic avian influenza virus (LPAIV) according to its pathogenicity. HPAIVs mainly consist of H5 or H7 subtypes [107], not only causing mortality in chickens, but also posing a serious threat to public health [108]. Similar to humans, avian IAV RNA is primarily sensed by TLR3 and TLR7 [109,110,111]. It was reported that the TLR3 and IFN-β mRNA levels were significantly upregulated in the brain, spleen, and lungs in H5N1-infected chickens [112]. Meanwhile, the same change was observed under the treatment by poly (I:C) in chickens [112,113,114]. Furthermore, homologs of several key proteins in mammalian TLR3 signal transduction pathway were also been identified in chickens, such as JNK, TRIF, TBK1, IκκE, etc. [111,115]. Thus, chicken TLR3 (chTLR3) may act as the avian IAV RNA sensor in chickens, performing similar functions as mammalian TLR3. However, the TLR3 signaling cascade in chickens has not been systematically characterized. Due to the gene sequence of chTLR8 was highly disrupted in chickens [116], instead, chTLR7 may play a major role in the recognition of avian IAV ssRNA in endosomes [116,117]. It was found that the expression of IL-Iβ is highly upregulated upon the stimulation of synthetic ssRNA, a chTLR7 ligand, leading to the antiviral response in vitro [118]. It was also found that the mRNA level of IL-Iβ was significantly upregulated by the treatment of chTLR7 agonist, while the IFN-I mRNA level had no change [116], which suggests that chTLR7 prefers to induce the production of proinflammatory cytokines rather than IFN-I. Since RIG-I is congenitally deficient in chickens [119], thus dsRNA in the cytoplasm is mainly recognized by chMDA5, which may account for the high susceptibility of chickens to avian IAV infection [120,121,122]. It was reported that avian IAV RNA was mainly sensed by chMDA5, which further activates IRF7 and NF-κB signaling pathways through interacting with chSTING, initiating IFN response [123,124]. Of note, due to the natural absence of IRF3 [111], the innate immune response is mainly mediated by IRF1 and/or IRF7 in chickens [125,126,127]. In addition, chicken DDX3X (chDDX3X) could also induce IFN-β production through the chDDX3X-chSTING-chIRF7 signaling pathway [128]. Thus, it seems that the RLRs signal transduction in chickens is highly dependent on chMDA5 and chSTING. Unfortunately, few reports are currently available regarding the formation of inflammasome induced by avian IAV RNA in chickens. More efforts will be required to investigate avian IAV-induced pyroptosis in the future.

### 2.3. The Innate Immune Response to IAV Infection in Other Species

In addition to humans and chickens, IAV also replicates in other species. In comparison with chickens, the homolog of RIG-I was present in ducks, which mediates innate antiviral responses through its CARD domain [129,130]. It was reported that RIG-I and MDA5 were all involved in the recognition of avian IAV RNA in ducks and then elicited the innate immune response through NF-κB or IRF7 signaling pathways [131,132,133]. In addition, TLR7 and TLR3 were also significantly activated upon avian IAV infection [134]. It seems that the RNA sensors in ducks are more complete than that of chickens, which may explain the higher resistance of ducks to avian IAV infection than chickens. Swine, another host for IAV, play an important role in IAV transmission and mutation, which results in a risk of bidirectional infection between humans and swine [135]. In particular, the outbreaks of the H1N1, a mutant strain in swine, have posed a severe threat to human health [136]. Thus, it is necessary to evaluate the innate immune response caused by IAV in swine. It was found that the expressions of TLR3, TLR7 and RIG-I in porcine alveolar macrophages were up-regulated during IAV infection, and the JAK-STAT and MAPK signaling pathways were highly activated [137,138]. However, MDA5 seems to contribute little to the recognition of IAV RNA in swine [139].

### 2.4. Regulation of the Innate Immune Response by MiRNAs and Viral Proteins upon IAV Infection

It was found that varied miRNAs were involved in the innate immune response upon IAV infection in humans [140]. On the one hand, some miRNAs play a positive role, inducing an antiviral immune response and inhibiting IAV replication. For example, the expression of miR-93 in alveolar epithelial cells infected with IAV could be significantly down-regulated, resulting in the release of the target protein JAK, which mediated innate immune response through the JAK-STAT signaling pathway [141]. The expression of miR-340 was down-regulated in IAV-infected A549 cells, relieving the inhibition of RIG-I and OAS2 and enhancing the innate immune response [142]. On the other hand, the other miRNAs play a negative role in innate immune response upon IAV infection. For instance, miR-146a-5p is up-regulated in human nasal epithelial cells (HNECs) infected with H3N2, and negatively regulates TRAF6 expression [143]. miR-29c enhances the expression of A20 protein induced by IAV, inhibiting the activity of NF-κB and the expression of pro-inflammatory cytokines [144]. Previous reviews have well documented the multiple roles of miRNAs in the regulation of innate immunity upon IAV infection [140,145]. In addition to miRNAs, other noncoding RNAs such as long non-coding RNAs (lncRNAs), and circular RNAs (circRNAs) may also involve regulation of mRNAs in control of gene transcription. Furthermore, some IAV viral proteins, as listed in Table 1, were also found directly suppress the innate immune response in different species.

**Table 1:** The roles of IAV proteins in innate immune response across the species.

Host	Proteins	Protein Function	References
Human	NS1	Inhibit the activation of RIG-I	[146]
		Inhibit the ubiquitination of RIG	[147,148,149]
		Inhibit the formation of NLRP3 inflammasome	[150]
		Bind with IKK and Inhibit the activation of NF-κB	[151,152]
		Inhibit the ubiquitination of TRAF3	[153]
		Degradation of sphingosine 1-phosphate lyase (SPL) and suppress IKKϵ-mediated type I IFN response	[154]
	PB1-F2	Destroy the mitochondrial membrane	[155,156]
		Impair innate immunity by inducing mitophagy	[157]
		Interact with IKKβ and Inhibit the activation of NF-κB	[158]
		Interact with IRF3 and reduced the expression of IFN-β	[159]
	PB1	Degrade the MAVS by autophagy	[160]
	PA-X	Degrade viral dsRNA	[161]
		Inhibit the RIG-I-MAVS signaling pathway	[162]
		Inhibit Ankrd17-mediated immune response	[163]
		inhibit NF-κB transcription	[164]
Chicken	NS1	Act in concert with polymerase complexes	[165]
	PB1-F2	Interact with MAVS and inhibit the IFN response	[166]
Duck	NS1	Inhibit the MDA5-mediated signaling pathway	[133]
	PB1-F2	Inhibit RIG-I ubiquitination	[167]
Swine	NS1	Impair ASC speck formation and inhibit IL-1β production	[168]

## 3. Coronavirus

Coronavirus is a linear single-stranded RNA virus, belonging to the genus of *Corona-virus* in the family *Coronaviridae* [169]. According to the phylogeny, it can be classified into four genera: α, β, γ, and δ [170]. Coronavirus can infect a variety of mammals and poultry, including humans. At present, severe respiratory symptoms are consistent in humans and poultry, while distinct gastrointestinal symptoms are present in swine [171]. Therefore, it remains a high demand to explore the innate immune response upon coronavirus infection. The innate immune response to coronavirus infection in different species is reviewed as follows.

### 3.1. The Innate Immune Response to Coronavirus Infection in Humans

Up to now, three types coronavirus pose a severe threat to public health, namely SARS-CoV, SARS-CoV-2 and MERS-CoV, all of which belong to β-Coronavirus [170,172,173]. Both SARS-CoV and SARS-CoV-2 have caused global pandemics, especially SARS-CoV-2, which has caused millions of people deaths and uncountable economic losses worldwide since its outbreak in late 2019 [169,174]. With the constant evolution of SARS-CoV-2, a variety of evolved strains such as the Delta mutant strain [175] and Omicron mutant strain [176] have emerged, which results in enormous challenges for its prevention and control. Previous studies reported that SARS-CoV inhibited the activation of IFN-I promoter in the early stage after infection [177,178], and SARS-CoV-2 showed a similar effect [179]. The early immunosuppression in host could benefit the proliferation of the coronavirus, leading to delayed production of inflammatory cytokines and causing serious clinical symptoms [177,180].

Similar to IAV, the RNA sensors for coronavirus are TLRs and RLRs [181,182]. In vivo, TLR3^−/−^ or TRIF^−/−^ mice showed an increased susceptibility to SARS-CoV infection compared to that of wild-type (WT) mice, displaying a more severe lesion in the lung and a higher viral titer in the serum than the WT mice [183]. It was, not until recently, found that TLR3 and TLR7, as direct intracellular RNA sensors, were highly activated in SARS-CoV-2-infected CalU-3/MRC-5 multicellular spheroids in the early stage of infection. Particularly, TLR3 activates the expression of IL-1β, IL-4, IL-6 and IFN-I largely through the IRF3 signaling pathway within 48 h after SARS-CoV-2 infection, while IRF7 seems to induce the expression of IFN-I and IFN-III through the activation of NF-κB after 48 h of viral infection [184]. These findings provide direct evidence that TLR3 acts as an RNA sensor of coronavirus. In addition, it was reported that severe cases with SARS-CoV-2 infection have more multiple mutations in TLR3 than those with mild clinical symptoms, and these mutations are closely related to the severity of clinical symptoms, suggesting that the mutations of TLR3 may be the potential reason for severe clinical symptoms [185]. Furthermore, it was found that the polymorphism of L412F in TLR3 was more closely related to the severity of SARS-CoV-2 infection [186]. These findings indicate a critical role of TLR3 in recognizing the coronavirus RNA, triggering an innate immune response to SARS-CoV-2 infection.

It was found that TLR7/8 played an indispensable role in the recognition of corona-virus ssRNA, triggering host response [187,188]. SARS-CoV ssRNA could be engaged by TLR7 in plasmacytoid dendritic cells (pDCs), eliciting a rapid innate immune response to suppress viral replication [189]. Furthermore, it was also found that TLR7 functioned as a primary sensor for viral ssRNA upon MERS-CoV infection, modulating the IFN-I response [190]. In early 2020, SARS-CoV-2 broke out worldwide [174]. In order to better understand the innate immune response caused by SARS-CoV-2, bioinformatics analysis was used to identify the ssRNA sequences sensed by TLR7/8 in the whole genomes of SARS-CoV-2, SARS-CoV and MERS-CoV. It was found that a number of viral ssRNA fragments could be sensed by TLR7/8, which provides coronavirus with a shortcut to trigger an innate immune response [191]. It should be noted that more UU (U/C) and UU (G/A) motifs existed in SARS-CoV-2 genomes compared with that of SARS-CoV, which may lead to increased inflammatory response and aggravated severe clinical symptoms [187]. Clinically, the patients, especially males, with TLR7 loss of function variants showed higher susceptibility to SARS-CoV-2 and lower expression level of IFN-I and IFN-II [192], while mutations in TLR8 seems to have no effect on its receptor function [193]. Taken together, lines of evidence show that both TLR7 and TLR8 play an essential role in sensing coronavirus ssRNA to initiate the innate immune response, and the mutations of TLR7 may be used as an indicator to evaluate the human susceptibility to SARS-CoV-2. Of note, since the genes encoding TLR7 and TLR8 are present on the X chromosome, sex preference occurred upon coronavirus infection, especially SARS-CoV-2, which is manifested by the low expression level of IL-6 and relatively mild clinical symptoms in women [194,195].

Different from IAV, coronavirus genome in the cytoplasm of host cells is primarily sensed by MDA5 and LGP2, consequently activating innate immune response [196,197,198]. It was found that SARS-CoV-2 replication intermediates specifically activated a delayed IFN-I response through MDA5, LGP2 and NOD1 in CalU 3 cells, while RIG-I did not affect IFN-I response [196]. Surprisingly, NOD1, a well-known sensor for bacterial peptidoglycans, was identified as a PRR sensing SARS-CoV-2 [196]. It would be interesting to explore the mechanism by which NOD1 triggers innate immune response upon SARS-CoV-2 infection. As for RIG-I, another important dsRNA sensor in the cytoplasm, its role in the innate immune response during SARS-CoV-2 infection remains controversial. It was early found that RIG-I had no contribution to IFN-β production upon SARS-CoV-2 infection [196,199], while a recent study indicated that the IFN-β expression significantly decreased in RIG-I^−/−^ CalU 3 cells [200]. Furthermore, it was reported that the RIG-I helicase domain (HD) was able to directly interact with the 3′UTR of SARS-CoV-2 RNA in human alveolar epithelial cells, and inhibited viral replication by blocking the binding of RNA-dependent RNA polymerase (RdRp) to viral RNA [201], suggesting that RIG-I plays a role in the host response to SARS-CoV-2 infection. Thus, it seems that RIG-I inhibits viral replication in an IFN-independent manner upon SARS-CoV-2 infection.

Evidence has shown that severe forms of SARS-CoV or SARS-CoV-2 infections were closely related to acute severe inflammatory reactions [202,203]. Multiple proinflammatory cytokines and inflammasome derivatives, such as IL-1β, IL-18, NLRP3 and LDH, were detected in the serum of patients infected with SARS-CoV-2, which suggests that a strong inflammatory response caused by SARS-CoV-2 may be closely related to inflammasome formation [203,204,205]. Persistent activation of NLRP3 inflammasomes can lead to severe clinical symptoms, including fever, necrosis, and severe inflammatory responses [206]. A recent study analyzed the inflammasome activation in the serum of 129 SARS-CoV-2 patients, and found that the expression of caspase-1 p20 and IL-18 in the serum was significantly elevated [207]. Furthermore, the NLRP3-ASC complex was observed in peripheral blood mononuclear cells (PBMCs), which indicates that NLRP3 is highly activated upon SARS-CoV-2 infection [207]. Moreover, a considerable amount of cleaved GSDMD, an indicator of pyroptosis, was also observed using multiplex immunohistochemistry [208]. These results indicate that SARS-CoV-2 is capable of initiating NLRP3 activation. Nevertheless, the effect of coronavirus RNA on the formation of inflammasomes is still not clear. It was, not until recently, found that GU-rich single-stranded RNA (GU-rich RNA) derived from SARS-CoV or SARS-CoV-2 could initiate a TLR8-dependent pro-inflammatory response in human macrophages without pyroptosis. GU-rich RNA can be engaged by TLR8 and activates inflammasomes through the TLR8-Caspase 8-RIPK3-NLRP3 signaling pathway, and releases mature IL-1β, IL-6 and TNF, which provides preliminary data favoring the involvement of coronavirus RNA in inflammasome formation [209,210] Further studies are required to determine whether coronavirus RNA triggers inflammatory responses through the classical pyroptotic pathway. Given the current pandemic of SARS-CoV-2, to better understand the innate immune recognition of coronaviruses with cross-species transmission capability, we comprehensively compared the innate sensing of three coronaviruses, MERS-CoV, SARS-CoV, and SARS-CoV-2 (Table 2).

**Figure 3:**
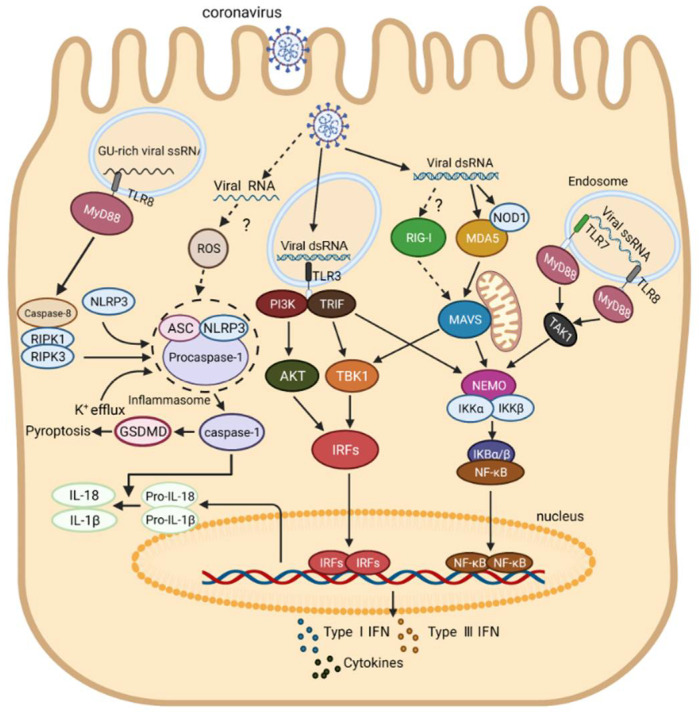
Schematic diagram of innate immune response induced by coronavirus RNA. Viral dsRNA is sensed by TLR3 in endosomes, triggering the innate immune response, while ssRNA in endosomes is sensed by TLR7/8 and induces the production of inflammatory cytokines through the MyD88-TAK1-IKK-NF-κB signaling pathway. Viral dsRNA is recognized by MDA5 and NOD1, and then interacts with MAVS on mitochondria, which further activates the NF-κB and IRFs signaling pathways. Viral GU-rich ssRNA is sensed by TLR8, causing the activation of NLRP3 inflammasomes through the Caspase-8-RIPK1 signaling pathway, leading to the secretion of mature IL-1β and IL-18. Importantly, it is not clear whether viral dsRNA is recognized by RIG-I to initiate IFN response or whether viral RNA induces inflammasome formation; thus, the associated pathway is indicated by dotted lines. Abbreviation: RIPK, Receptor interacting protein kinase; other abbreviations are shown in the legend to Figure 2.

Interestingly, it was found that the cGAS-STING signaling pathway, a signaling pathway activated by recognition of DNA by cellular cGAS, was also activated to drive a robust IFN-I response after SARS-CoV-2 infection [218,219]. Different from the direct sense for viral RNA by TLRs and RLRs, cGAS-STING activation mainly relies on the recognition of DNA from either pathogens or host tissue damages [220]. RNA sensors-mediated signal transduction pathways in the innate immune response to coronavirus infection in humans are summarized and shown in Figure 3.

### 3.2. The Innate Immune Response to Coronavirus Infection in Chickens

Avian infectious bronchitis virus (IBV), a representative avian coronavirus, mainly causes respiratory symptoms, but the lack of available cell lines for viral infection has limited its further investigation [221]. Thus, the current research on IBV is mainly focused on vivo experiments. At present, due to the emergence of mutant strains, the control of IBV is becoming more and more difficult [222]. An in-depth understanding of the innate immune response to IBV infection is very important. Existing evidence suggests that chTLR3, chTLR7, and chMDA5 are involved in the recognition of IBV RNA to trigger the innate immune response [223]. Upon IBV M41 strain infection, the TLR3-TRIF signaling pathway was highly activated, which further triggered the innate immune response and inhibited IBV replication [224,225]. In contrast, it was found that the mRNA level of chTLR3 was down-regulated in respiratory epithelial cells early during IBV Connecticut strain infection [226]. Furthermore, the effect of IBV infection on the chTLR7 signaling pathway is still not very clear [227]. It was reported that the expression of chMDA5 and chSTING were up-regulated in IBV-infected birds, which further initiated the innate immune response [225,228]. chLGP2, a member of RLRs with less investigated in chicken, was reported to interact with chTRBP to dampen IBV infection [229]. The research on the innate immune response to coronavirus infection in chickens is quite limited and should be highly encouraged.

### 3.3. The Innate Immune Response to Coronavirus Infection in Other Species

Porcine coronavirus consists of porcine respiratory coronavirus (PRCV), porcine delta coronavirus (PDCoV), porcine epidemic diarrhea virus (PEDV), transmissible gastroenteritis virus (TGEV), etc. It was reported that viral RNA could be sensed by TLR3 and TLR9 to activate the NF-κB signaling pathway in PEDV-infected IECs, which is independent of RIG-I [230]. Meanwhile, the mRNA levels of MDA5 were significantly up-regulated in TGEV-infected porcine kidney cells, promoting the expression of inflammatory cytokines [231]. Of note, a recent study found that PDCoV infection robustly activated the expression of RIG-I, thus enhancing the IFN-β production [232]. It was also demonstrated that TLR7 expression was significantly upregulated and elicited IFN response after PEDV infection in IPEC-J2 cells [233]. Furthermore, it was recently found that NLRP3 was highly activated in porcine coronavirus infected cells, leading to pyroptosis as demonstrated by cleaved GSDMD and IL-1β release [234], suggesting that NLRs may play an important role in the innate immune response to porcine coronavirus infection.

### 3.4. The Innate Immune Regulation by MiRNAs and Viral Proteins during Coronavirus Infection

Up to now, there are few reports regarding the regulation of innate immune response by miRNAs during coronavirus infection in humans. It was found that miRNAs were involved in innate immune response, viral binding, viral invasion, and intracellular stress after coronavirus infection [235,236,237,238]. Angiotensin-converting enzyme 2 (ACE2), which serves as a receptor for coronavirus, can interact with transmembrane serine protease 2 (TMPRSS2) to facilitate viral entry [239]. It was reported that miR-200c could target the 3′-UTR of ACE2 mRNA, reducing the ACE expression and suppressing viral entry [240]. Similar to miR-200c, miR-98-5p suppressed the expression of TMPRSS2 [241]. Interestingly, a recent study identified four unique microRNA-like small RNAs encoded by SARS-CoV-2, namely SCV2-miR-1ab-1-3p, SCV2-miR-1ab-2-5p, SCV2-miR-1ab-3-5p, and SCV2-miR-3a-5p, which targets the gene of IFN-I signaling pathway, regulating innate immunity [242]. Furthermore, two virus-derived miRNA isoforms were identified in SARS-CoV-2-infected human cells, namely CoV-2-miR-O7a.1 and CoV-2-miR-O7a.2. CoV-2-miR-O7a.2 suppresses ISGs expression by targeting its 3′UTR and evades the IFN-mediated immune response [243]. Except for the limited research, current research performed a functional enrichment analysis regarding the roles of miRNAs in the inflammatory response in SARS-CoV-2-infected patients. Specifically, three miRNAs were significantly down-regulated in SARS-CoV-2-infected patients, namely miR-26a-5p, miR-29b-3p, and miR-34a-5p. Among them, miR-26a-5p may regulate inflammatory response by targeting IL-6, and miR-34a-5p is predicted to regulate inflammation by targeting Caspase-1 [244].

In contrast to miRNAs, the roles of coronavirus proteins in the innate immune response have been widely investigated across the species (Table 3).

## 4. The Differences in Innate Immune Response between Species during IAV/Coronavirus Infections

Innate immune response is highly different between species for both IAV and coronavirus infections. On the one hand, the differences in PRRs between species directly influence the host’s innate immune recognition. For instance, the congenital deficiency of RIG-I in chicken enhances its susceptibility to these two viruses greatly [119]. On the other hand, the discrepancy in protein-protein interactions (PPIs) modulated by innate immune recognition of viral RNA also contributes to the differences in innate immune responses across the species. It was reported that the MAVS-IRF3 signaling pathway plays an important role in innate immune response in humans [292], whereas in chickens, it seems to be more inclined to induce innate immune response via the STING-IRF7/MAVS-IRF7 signaling pathway [128]. Damaged dsDNA could be recognized by TLR9/cGAS upon coronavirus infection, thereby inducing an innate immune response via the TLR9-MyD88/cGAS-STING signaling pathway in humans [220,293], but similar mechanism has not been identified in chickens. In addition, it was found that NOD1, an RNA sensor for corona-virus [196], could recognize poly (I:C) dsRNA and induce an innate immune response by binding to MDA5 in humans [294], whereas no similar report has been found in other species. Furthermore, a recent study found that PDCoV infection induced the expression of IFN-β via the RIG-MAVS signaling pathway in swine [232], however, RIG-1 initiates host response in an IFN-independent way by competitively binding to the coronavirus RNA with RdRp in humans [201].

## 5. Conclusions

Up to now, IAV and coronavirus have posed a serious threat to animals and public health. As the first line of host defense against virus infection, innate immune response is particularly important. TLRs, NLRs, and RLRs play pivotal roles in innate immune response via recognizing IAV and coronavirus RNAs to trigger innate immune signaling pathways. Both IAV and coronavirus RNAs in endosomes are sensed by TLR3 and TLR7. However, IAV appears to trigger the innate immune response mainly through RIG-I, whereas coronavirus prefers to rely on MDA5. It is still unclear whether there are any RNA sensors currently unknown to us and are involved in the innate immune response to IAV and coronavirus infections. However, the innate immune recognition of IAV/coronavirus RNA also varies considerably across species, including the differences in PRRs and PPIs modulated by innate immune recognition of viral RNA. This review primarily focuses on PRRs-mediated innate immune signaling and provides an overview of the roles of RNA sensors in host innate response to IAV and coronavirus infections in different species. A comprehensive understanding of the mechanisms by which these two respiratory viral pathogens initiate the innate immune response via RNA sensors will help us better understand the difference in susceptibility across the species and develop novel vaccines or antiviral drugs for their effective control.

## Figures and Tables

**Figure 2:**
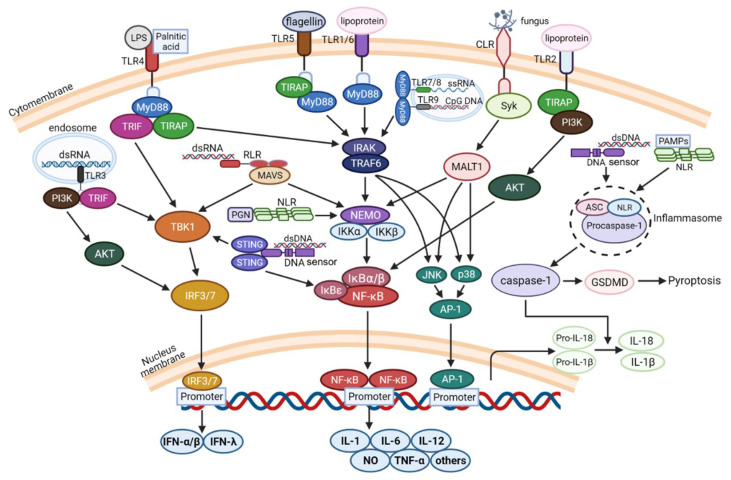
Schematic diagram of signaling transduction pathways in host response induced by recognition of PAMPs by PRRs. Sensing PAMPs, the PRRs recruit and activate MyD88, TRIF, TIRAP, and other adapters. These adapters transduce activating signals, which finally induce activation of transcriptional regulators NF-κB and IRFs, leading to the expression of IFNs and proinflammatory cytokines or mediators, such as IL-1, IL-6, IL-12, TNF-α, NO, etc. Abbreviation: MyD88, myeloid differentiation factor 88; TRIF, TIR domain-containing adapter-inducing IFN-β; TIRAP, TIR domain-containing adapter protein; IRAK, IL-1 receptor kinase; TRAF, tumor necrosis factor receptor-associated factor; MALT1, mucosa-associated lymphoid tissue lymphoma translocation gene 1; Syk, spleen tyrosine kinase; TAK, TGF-β activated kinase; NEMO, NF-κB essential modulator; IKK, inhibitor of NF-κB kinase; IκB, inhibitor of NF-κB; MAVS, mitochondrial antiviral signaling protein; PI3K, phosphoinositol-3 kinase; NF-κB, nuclear factor kappa enhancer binding protein; IRF, interferon regulatory factor; RIG-I, retinoic acid-inducible gene I; MDA5, melanoma differentiation-associated gene 5; STING, stimulator of interferon genes; LPS, Lipopolysaccharide; GSDMD, gasdermin D.

**Table 2:** The comparison of innate immune sensors involved in recognition of MERS-CoV, SARS-CoV, and SARS-CoV-2 infections.

Virus	TLRs	RLRs	NLRs	CLR	Others
MERS-CoV	TLR7 [190] TLR8 [191]	RIG-I [211] MDA5 [211]	NLRP3 [212]	Mincle [211]	PKR [213]
SARS-CoV	TLR7 [189] TLR8 [191] TLR4 [183] TLR2 [214]	N/A ^1^	NLRP3 [215]	N/A	N/A
SARS-CoV-2	TLR3 [184] TLR7 [184] TLR8 [191] TLR4 [216] TLR2 [201]	MDA5 [196] LGP2 [196] RIG-I [201]	NOD1 [196] NLRP3 [207]	N/A	PKR [217] OAS [217]

^1^ N/A: not applicable (data for the corresponding sensor has not been reported).

**Table 3:** The roles of coronavirus proteins in innate immune response across the species.

Host	Virus	Viral Protein	Protein Function	References
Human	SARS-CoV	M	Delay the formation of TRAF3-containing complex	[245]
		N	Inhibit the ubiquitination of RIG-I mediated by TRIM25	[246]
		N, ORF3b, ORF6	Inhibit the activation of IRF3 and the translocation of NF-κB	[247]
		NSP1	Decrease the phosphorylation of STAT1	[178]
		PLpro(Nsp3)	Inhibit the ubiquitination of STING, TBK1 and IRF3	[248,249]
		Nsp14, Nsp16, Nsp10	Induce viral mRNA cap methylation and escape the detection of MDA5	[250,251]
		ORF3a	Degrade the expression of IFNAR1	[252]
		ORF6	Inhibit the translocation of STAT1	[253]
	SARS-CoV-2	M	Degrade the ubiquitinated TBK1 and inhibit IFN-I response	[254]
		N	Interact with RIG-I and suppress the RIG-I signaling pathway	[255]
		Nsp1	Bind to mRNA entry channel of the ribosome and inhibit ISGs mRNA translation	[256]
		Nsp5	Cleave RIG-I and promote the proteosome-mediated degradation of MAVS	[257]
		Nsp6	Inhibit the activation of IRF3 and suppress the phosphorylation of STAT1 and STAT2	[258]
		Nsp8, Nsp9	Disrupt the function of signal recognition particle (SRP) complex and suppress IFN trafficking	[256]
		Nsp16	Inhibit ISGs mRNA splicing and suppress innate immune response	[256]
		Nsp12	Inhibit the IRF3 nuclear translocation and attenuate IFN-I production	[259]
		Nsp13	Interact with TBK1 and inhibit its activation	[260]
		Nsp14	Shut down the protein synthesis and abolish the production of ISGs	[261]
		Nsp15	Interact with RNF41 and inhibit the activation of TBK1	[260]
		ORF3a	Interact with STING and inhibit the nuclear import of NF-κB	[262]
		ORF6	Interact with NUP98-RAE1 Inhibit STAT1 nuclear translocation	[260] [263]
		ORF7a	Destabilize the TBK1 and lead to attenuated IRF-3 phosphorylation	[264]
		ORF8	Induce ER stress and inhibit the nuclear translocation of IRF3	[265]
		ORF9b	Interact with RIG-I, MDA-5, and STING and inhibit the phosphorylation of IRF3	[266]
			Interact with TOM70 and suppress the innate immune response	[267]
			Interrupt the K63-linked polyubiquitination of NEMO and inhibit IKKα/β/γ-NF-κB signaling pathway	[268]
		ORF10	Degrade MAVS through mitophagy	[269]
	MERS-CoV	NS4a	Impede the activation of PKR	[213,270]
		NS4b	Inhibit the activation of RNaseL	[271]
			Bind to karyopherin-α4 (KPNA4) and inhibit the translocate of NF-κB	[272]
		ORF4a	Inhibit the activation of MDA5	[273,274]
		ORF4b	Inhibit the formation of TBK1 signaling complex	[275]
		ORF5	Inhibit the activation of NF-κB	[276]
Chicken	IBV	Nsp2	Inhibit the activation of PKR	[277]
		Nsp3	Interrupt the activation of RIG-I and NF-κB	[278]
		Accessory protein 5b	Induce a host shutoff and suppress IFN production	[279]
Swine	PEDV	Nsp1	Inhibit the activation of IRF1/IRF3	[280]
			Degrade CREB-binding protein and inhibit IFN-I production	[281]
			Inhibit the translocation of NF-κB	[282]
		ORF3	Inhibit the phosphorylation of IκBα and NF-κB translocation	[283]
		PLP (Nsp3)	Deubiquitinate RIG-I and STING	[284]
		3CLpro (Nsp5)	Cleave NEMO to inhibit the innate immune response	[285]
		Nsp15	RNA degradation of TBK1 and IRF3.	[286]
		Nsp16	Inhibit the activation of RIG-I and MDA5	[287]
		N	Inhibit the translocation of NF-κB	[288]
			Interrupt the interaction between IRF3 and TBK1	[289]
		E	Inhibit the RIG-I mediated IFN-β production	[290]
	TGEV	Nsp3	Inhibit the activation of NF-κB	[291]
